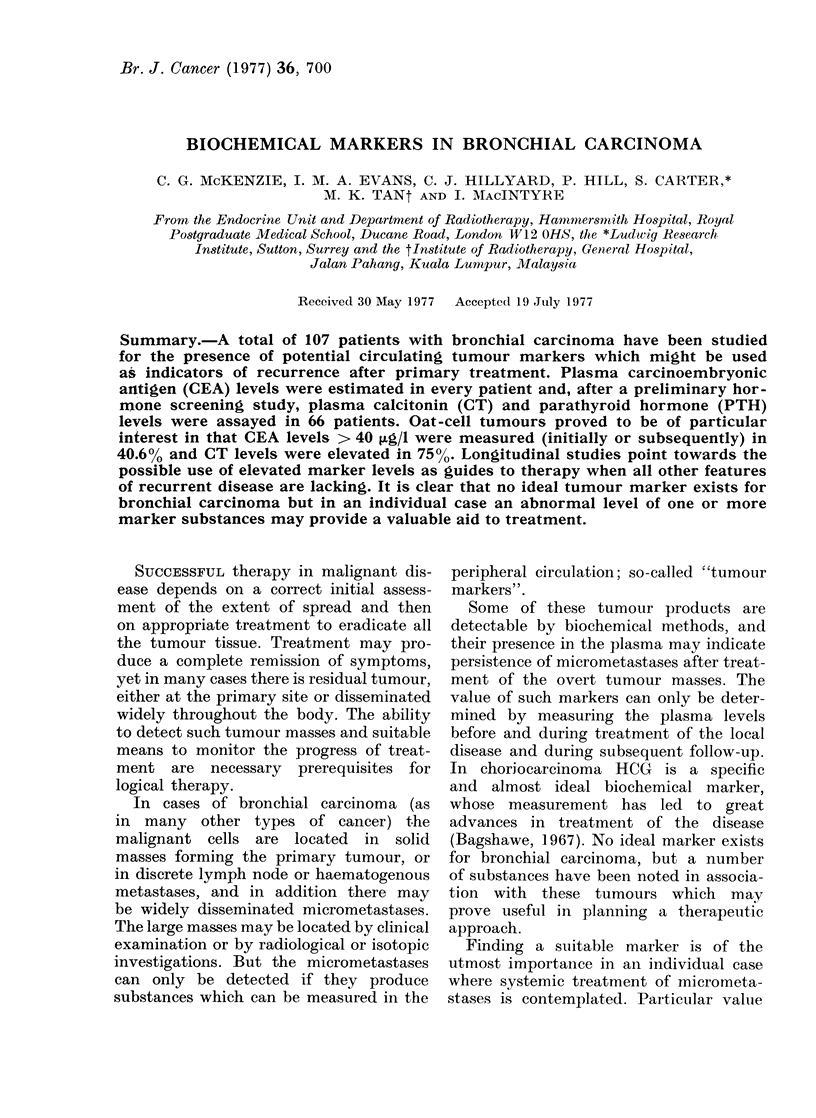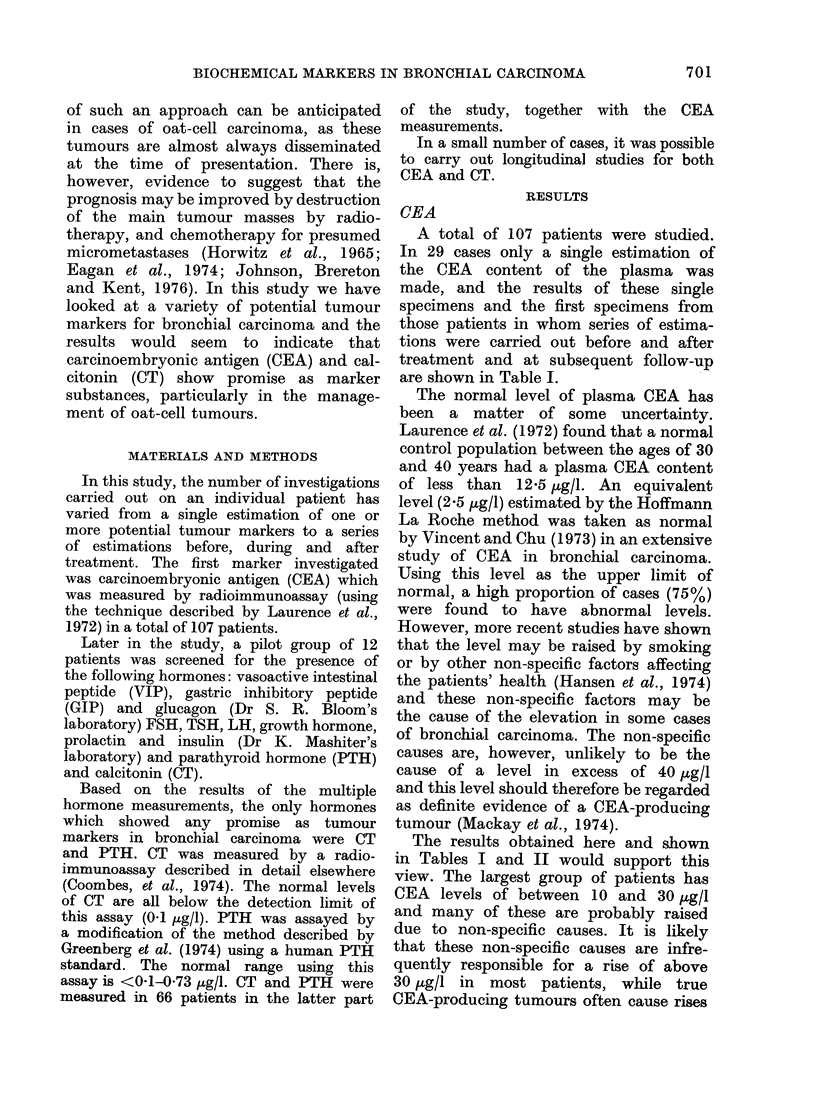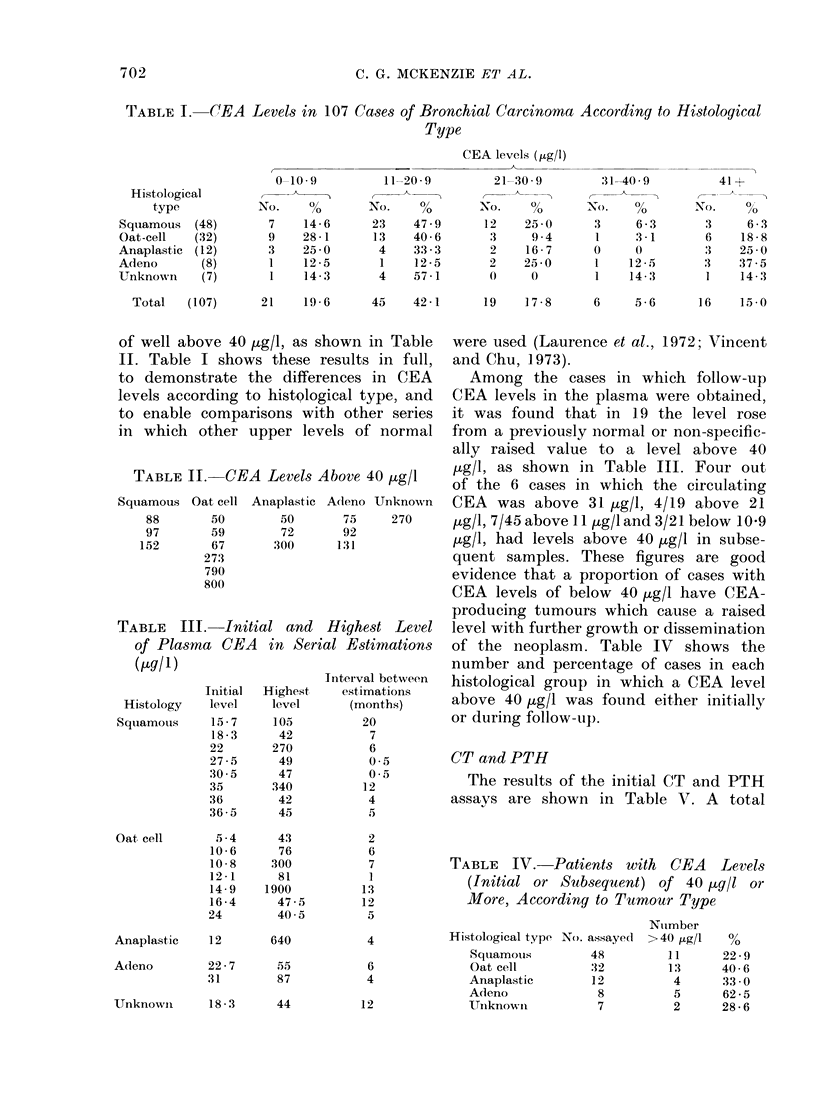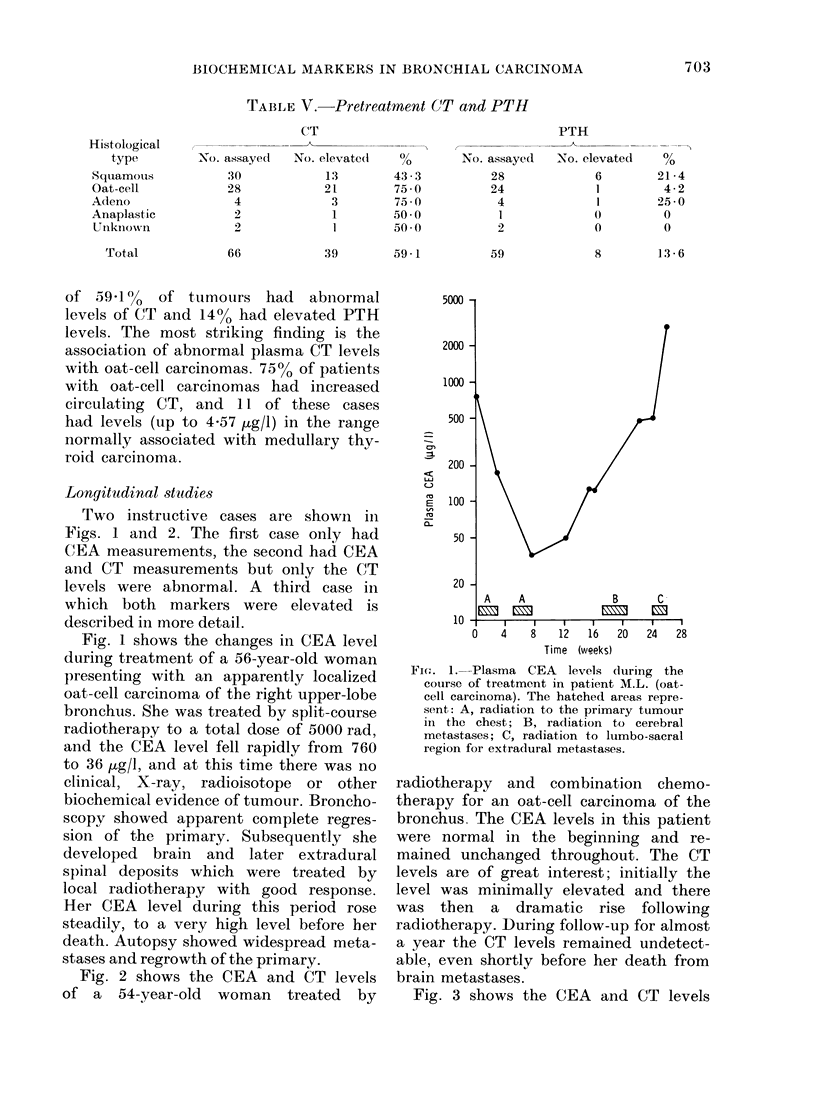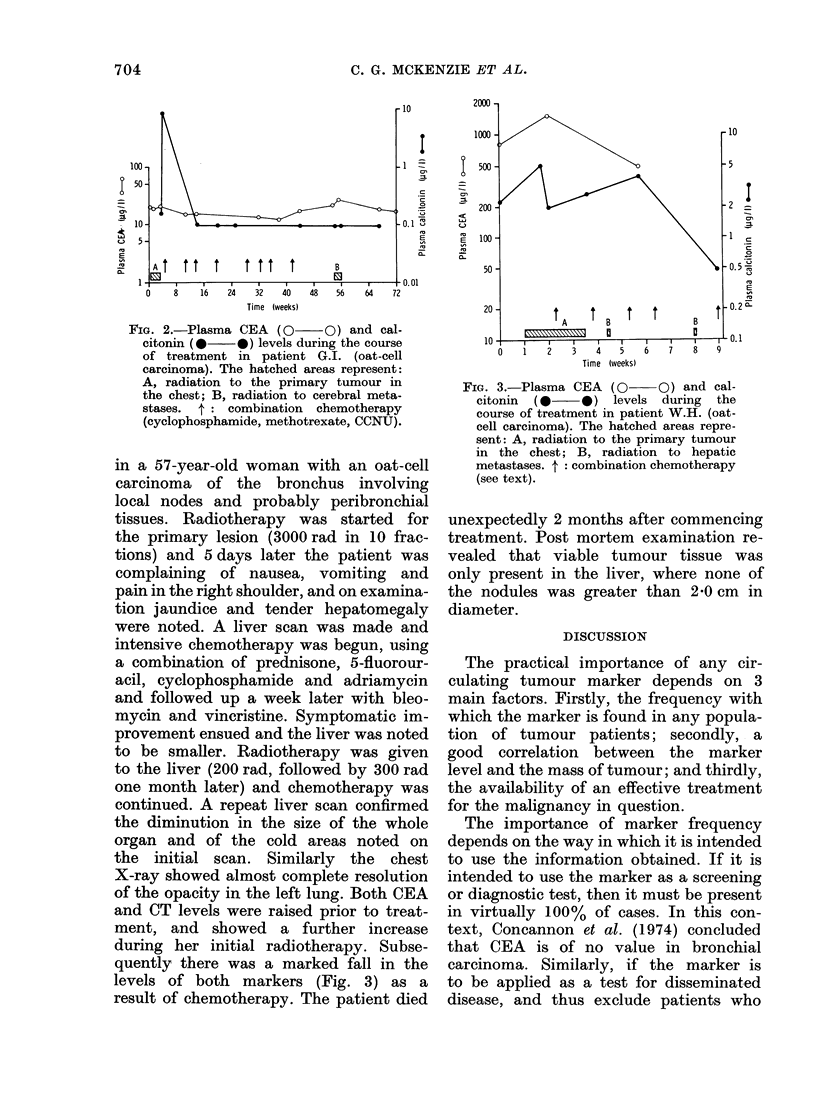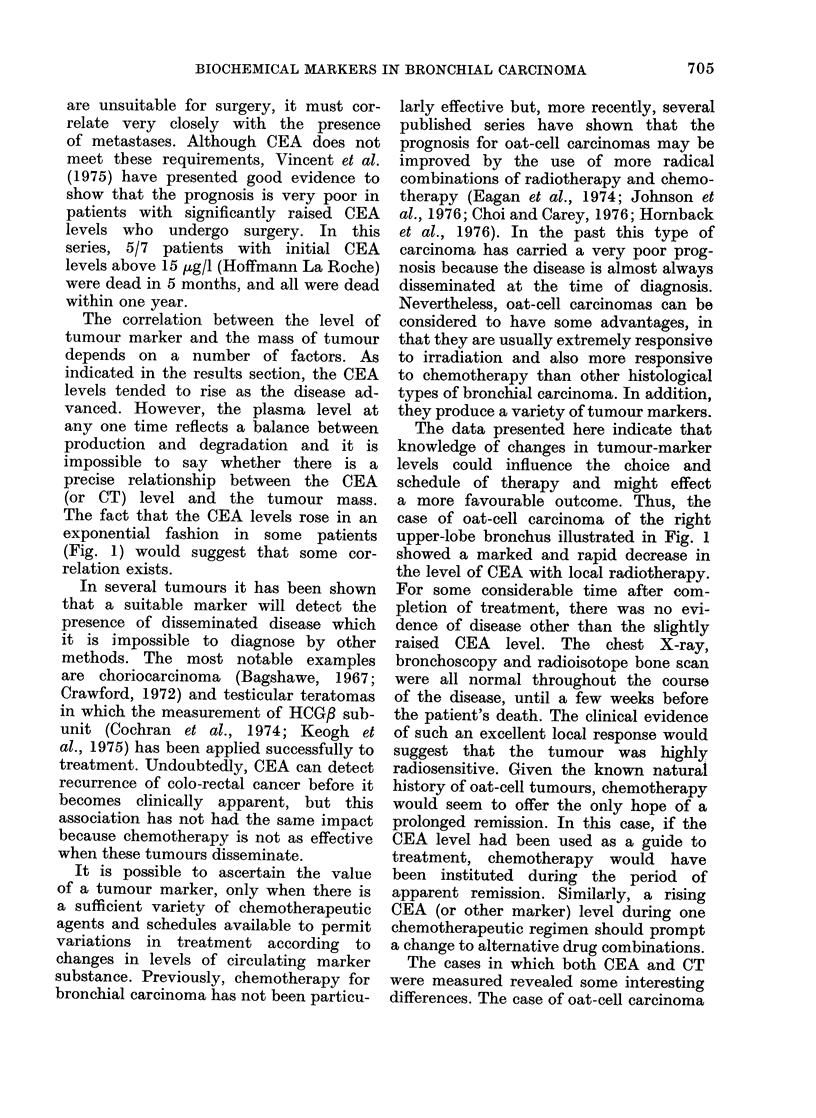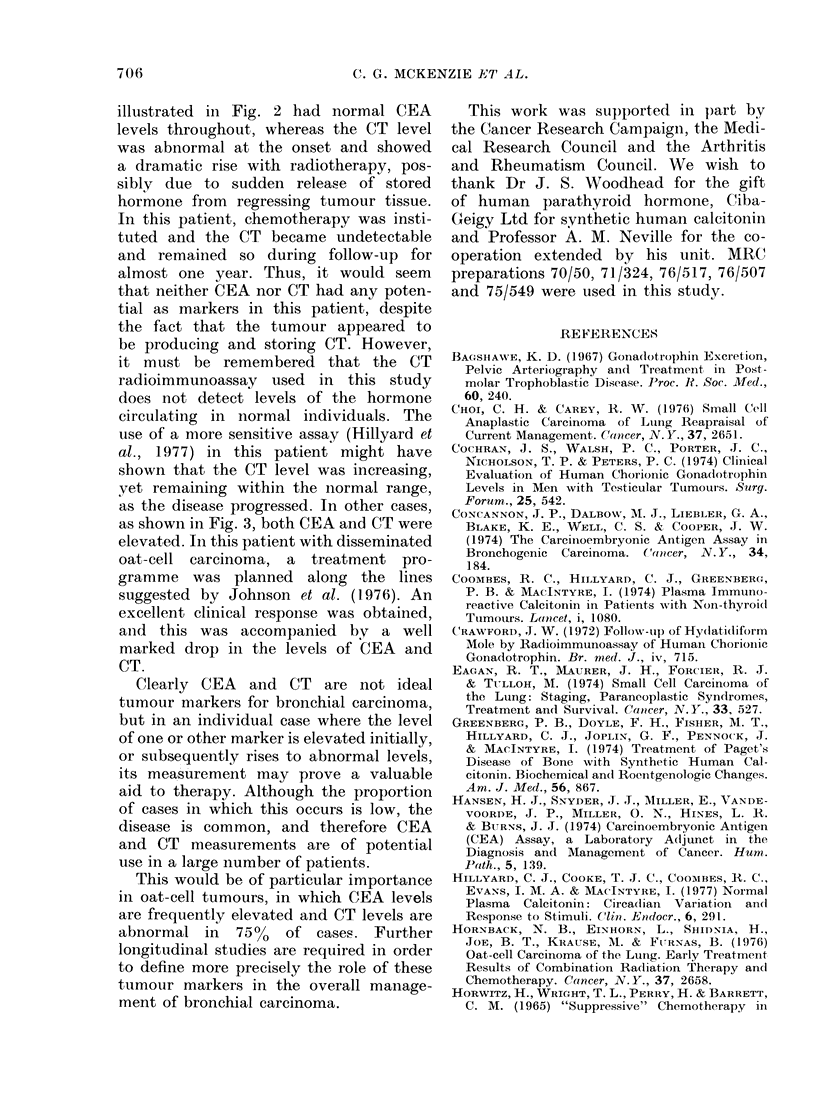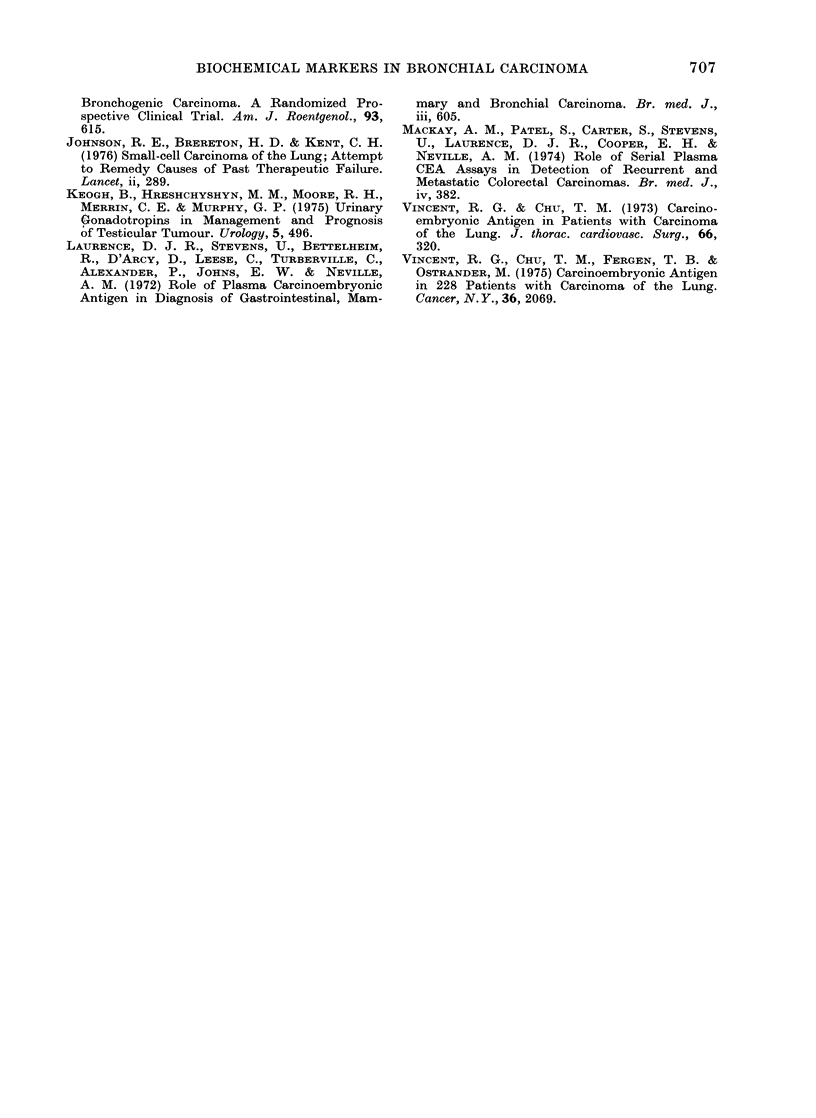# Biochemical markers in bronchial carcinoma.

**DOI:** 10.1038/bjc.1977.252

**Published:** 1977-12

**Authors:** C. G. McKenzie, I. M. Evans, C. J. Hillyard, P. Hill, S. Carter, M. K. Tan, I. MacIntyre

## Abstract

A total of 107 patients with bronchial carcinoma have been studied for the presence of potential circulating tumour markers which might be used as indicators of recurrence after primary treatment. Plasma carcinoembryonic antigen (CEA) levels were estimated in every patient and, after a preliminary hormone screening study, plasma calcitonin (CT) and parathyroid hormone (PTH) levels were assayed in 66 patients. Oat-cell tumours proved to be of particular interest in that CEA levels greater than 40 microgram/l were measured (initially or subsequently) in 40.6 percent and CT levels were elevated in 75 percent. Longitudinal studies point towards the possible use of elevated marker levels as guides to therapy when all other features of recurrent disease are lacking. It is clear that no ideal tumour marker exists for bronchial carcinoma but in an individual case an abnormal level of one or more marker substances may provide a valuable aid to treatment.


*Br. J. Cancer* (1977) **36**, 700

### BIOCHEMICAL MARKERS IN BRONCHIAL CARCINOMA

C. G. McKENZIE, I. M. A. EVANS, C. J. HILLYARD, P. HILL, S. CARTER,*
M. K. TAN† AND I. MacINTYRE

*From the Endocrine Unit and Department of Radiotherapy, Hammersmith Hospital, Royal Postgraduate Medical School, Ducane Road, London W12 0HS, the *Ludwig Research Institute, Sutton, Surrey and the †Institute of Radiotherapy, General Hospital, Jalan Pahang, Kuala Lumpur, Malaysia*

Received 30 May 1977 Accepted 19 July 1977

**Summary.—A total of 107 patients with bronchial carcinoma have been studied for the presence of potential circulating tumour markers which might be used as indicators of recurrence after primary treatment. Plasma carcinoembryonic antigen (CEA) levels were estimated in every patient and, after a preliminary hormone screening study, plasma calcitonin (CT) and parathyroid hormone (PTH) levels were assayed in 66 patients. Oat-cell tumours proved to be of particular interest in that CEA levels $> 40$ μg/l were measured (initially or subsequently) in 40.6% and CT levels were elevated in 75%. Longitudinal studies point towards the possible use of elevated marker levels as guides to therapy when all other features of recurrent disease are lacking. It is clear that no ideal tumour marker exists for bronchial carcinoma but in an individual case an abnormal level of one or more marker substances may provide a valuable aid to treatment.**

SUCCESSFUL therapy in malignant disease depends on a correct initial assessment of the extent of spread and then on appropriate treatment to eradicate all the tumour tissue. Treatment may produce a complete remission of symptoms, yet in many cases there is residual tumour, either at the primary site or disseminated widely throughout the body. The ability to detect such tumour masses and suitable means to monitor the progress of treatment are necessary prerequisites for logical therapy.

In cases of bronchial carcinoma (as in many other types of cancer) the malignant cells are located in solid masses forming the primary tumour, or in discrete lymph node or haematogenous metastases, and in addition there may be widely disseminated micrometastases. The large masses may be located by clinical examination or by radiological or isotopic investigations. But the micrometastases can only be detected if they produce substances which can be measured in the peripheral circulation; so-called "tumour markers".

Some of these tumour products are detectable by biochemical methods, and their presence in the plasma may indicate persistence of micrometastases after treatment of the overt tumour masses. The value of such markers can only be determined by measuring the plasma levels before and during treatment of the local disease and during subsequent follow-up. In choriocarcinoma HCG is a specific and almost ideal biochemical marker, whose measurement has led to great advances in treatment of the disease (Bagshawe, 1967). No ideal marker exists for bronchial carcinoma, but a number of substances have been noted in association with these tumours which may prove useful in planning a therapeutic approach.

Finding a suitable marker is of the utmost importance in an individual case where systemic treatment of micrometastases is contemplated. Particular value of such an approach can be anticipated in cases of oat-cell carcinoma, as these tumours are almost always disseminated at the time of presentation. There is, however, evidence to suggest that the prognosis may be improved by destruction of the main tumour masses by radiotherapy, and chemotherapy for presumed micrometastases (Horwitz *et al.*, 1965; Eagan *et al.*, 1974; Johnson, Brereton and Kent, 1976). In this study we have looked at a variety of potential tumour markers for bronchial carcinoma and the results would seem to indicate that carcinoembryonic antigen (CEA) and calcitonin (CT) show promise as marker substances, particularly in the management of oat-cell tumours.

### MATERIALS AND METHODS

In this study, the number of investigations carried out on an individual patient has varied from a single estimation of one or more potential tumour markers to a series of estimations before, during and after treatment. The first marker investigated was carcinoembryonic antigen (CEA) which was measured by radioimmunoassay (using the technique described by Laurence *et al.*, 1972) in a total of 107 patients.

Later in the study, a pilot group of 12 patients was screened for the presence of the following hormones: vasoactive intestinal peptide (VIP), gastric inhibitory peptide (GIP) and glucagon (Dr S. R. Bloom's laboratory) FSH, TSH, LH, growth hormone, prolactin and insulin (Dr K. Mashiter's laboratory) and parathyroid hormone (PTH) and calcitonin (CT).

Based on the results of the multiple hormone measurements, the only hormones which showed any promise as tumour markers in bronchial carcinoma were CT and PTH. CT was measured by a radioimmunoassay described in detail elsewhere (Coombes, *et al.*, 1974). The normal levels of CT are all below the detection limit of this assay (0·1 μg/l). PTH was assayed by a modification of the method described by Greenberg *et al.* (1974) using a human PTH standard. The normal range using this assay is <0·1–0·73 μg/l. CT and PTH were measured in 66 patients in the latter part of the study, together with the CEA measurements.

In a small number of cases, it was possible to carry out longitudinal studies for both CEA and CT.

### RESULTS

### *CEA*

A total of 107 patients were studied. In 29 cases only a single estimation of the CEA content of the plasma was made, and the results of these single specimens and the first specimens from those patients in whom series of estimations were carried out before and after treatment and at subsequent follow-up are shown in Table I.

The normal level of plasma CEA has been a matter of some uncertainty. Laurence *et al.* (1972) found that a normal control population between the ages of 30 and 40 years had a plasma CEA content of less than 12·5 μg/l. An equivalent level (2·5 μg/l) estimated by the Hoffmann La Roche method was taken as normal by Vincent and Chu (1973) in an extensive study of CEA in bronchial carcinoma. Using this level as the upper limit of normal, a high proportion of cases (75%) were found to have abnormal levels. However, more recent studies have shown that the level may be raised by smoking or by other non-specific factors affecting the patients' health (Hansen *et al.*, 1974) and these non-specific factors may be the cause of the elevation in some cases of bronchial carcinoma. The non-specific causes are, however, unlikely to be the cause of a level in excess of 40 μg/l and this level should therefore be regarded as definite evidence of a CEA-producing tumour (Mackay *et al.*, 1974).

The results obtained here and shown in Tables I and II would support this view. The largest group of patients has CEA levels of between 10 and 30 μg/l and many of these are probably raised due to non-specific causes. It is likely that these non-specific causes are infrequently responsible for a rise of above 30 μg/l in most patients, while true CEA-producing tumours often cause rises

TABLE I.—*CEA Levels in* 107 *Cases of Bronchial Carcinoma According to Histological Type*

| Histological type | | CEA levels (μg/l) 0–10·9 No. | % | 11–20·9 No. | % | 21–30·9 No. | % | 31–40·9 No. | % | 41+ No. | % |
|---|---|---|---|---|---|---|---|---|---|---|---|
| Squamous | (48) | 7 | 14·6 | 23 | 47·9 | 12 | 25·0 | 3 | 6·3 | 3 | 6·3 |
| Oat-cell | (32) | 9 | 28·1 | 13 | 40·6 | 3 | 9·4 | 1 | 3·1 | 6 | 18·8 |
| Anaplastic | (12) | 3 | 25·0 | 4 | 33·3 | 2 | 16·7 | 0 | 0 | 3 | 25·0 |
| Adeno | (8) | 1 | 12·5 | 1 | 12·5 | 2 | 25·0 | 1 | 12·5 | 3 | 37·5 |
| Unknown | (7) | 1 | 14·3 | 4 | 57·1 | 0 | 0 | 1 | 14·3 | 1 | 14·3 |
| Total | (107) | 21 | 19·6 | 45 | 42·1 | 19 | 17·8 | 6 | 5·6 | 16 | 15·0 |

of well above 40 μg/l, as shown in Table II. Table I shows these results in full, to demonstrate the differences in CEA levels according to histological type, and to enable comparisons with other series in which other upper levels of normal were used (Laurence *et al.*, 1972; Vincent and Chu, 1973).

TABLE II.—*CEA Levels Above* 40 μg/l

| Squamous | Oat cell | Anaplastic | Adeno | Unknown |
|---|---|---|---|---|
| 88 | 50 | 50 | 75 | 270 |
| 97 | 59 | 72 | 92 | |
| 152 | 67 | 300 | 131 | |
| | 273 | | | |
| | 790 | | | |
| | 800 | | | |

TABLE III.—*Initial and Highest Level of Plasma CEA in Serial Estimations* (μg/l)

| Histology | Initial level | Highest level | Interval between estimations (months) |
|---|---|---|---|
| Squamous | 15·7 | 105 | 20 |
| | 18·3 | 42 | 7 |
| | 22 | 270 | 6 |
| | 27·5 | 49 | 0·5 |
| | 30·5 | 47 | 0·5 |
| | 35 | 340 | 12 |
| | 36 | 42 | 4 |
| | 36·5 | 45 | 5 |
| Oat cell | 5·4 | 43 | 2 |
| | 10·6 | 76 | 6 |
| | 10·8 | 300 | 7 |
| | 12·1 | 81 | 1 |
| | 14·9 | 1900 | 13 |
| | 16·4 | 47·5 | 12 |
| | 24 | 40·5 | 5 |
| Anaplastic | 12 | 640 | 4 |
| Adeno | 22·7 | 55 | 6 |
| | 31 | 87 | 4 |
| Unknown | 18·3 | 44 | 12 |

Among the cases in which follow-up CEA levels in the plasma were obtained, it was found that in 19 the level rose from a previously normal or non-specifically raised value to a level above 40 μg/l, as shown in Table III. Four out of the 6 cases in which the circulating CEA was above 31 μg/l, 4/19 above 21 μg/l, 7/45 above 11 μg/l and 3/21 below 10·9 μg/l, had levels above 40 μg/l in subsequent samples. These figures are good evidence that a proportion of cases with CEA levels of below 40 μg/l have CEA-producing tumours which cause a raised level with further growth or dissemination of the neoplasm. Table IV shows the number and percentage of cases in each histological group in which a CEA level above 40 μg/l was found either initially or during follow-up.

### *CT and PTH*

The results of the initial CT and PTH assays are shown in Table V. A total

TABLE IV.—*Patients with CEA Levels (Initial or Subsequent) of* 40 μg/l *or More, According to Tumour Type*

| Histological type | No. assayed | Number >40 μg/l | % |
|---|---|---|---|
| Squamous | 48 | 11 | 22·9 |
| Oat cell | 32 | 13 | 40·6 |
| Anaplastic | 12 | 4 | 33·0 |
| Adeno | 8 | 5 | 62·5 |
| Unknown | 7 | 2 | 28·6 |

TABLE V.—*Pretreatment CT and PTH*

| Histological type | CT | | | PTH | | |
|---|---|---|---|---|---|---|
| | No. assayed | No. elevated | % | No. assayed | No. elevated | % |
| Squamous | 30 | 13 | 43·3 | 28 | 6 | 21·4 |
| Oat-cell | 28 | 21 | 75·0 | 24 | 1 | 4·2 |
| Adeno | 4 | 3 | 75·0 | 4 | 1 | 25·0 |
| Anaplastic | 2 | 1 | 50·0 | 1 | 0 | 0 |
| Unknown | 2 | 1 | 50·0 | 2 | 0 | 0 |
| Total | 66 | 39 | 59·1 | 59 | 8 | 13·6 |

of 59·1% of tumours had abnormal levels of CT and 14% had elevated PTH levels. The most striking finding is the association of abnormal plasma CT levels with oat-cell carcinomas. 75% of patients with oat-cell carcinomas had increased circulating CT, and 11 of these cases had levels (up to 4·57 μg/l) in the range normally associated with medullary thyroid carcinoma.

### *Longitudinal studies*

Two instructive cases are shown in Figs. 1 and 2. The first case only had CEA measurements, the second had CEA and CT measurements but only the CT levels were abnormal. A third case in which both markers were elevated is described in more detail.

Fig. 1 shows the changes in CEA level during treatment of a 56-year-old woman presenting with an apparently localized oat-cell carcinoma of the right upper-lobe bronchus. She was treated by split-course radiotherapy to a total dose of 5000 rad, and the CEA level fell rapidly from 760 to 36 μg/l, and at this time there was no clinical, X-ray, radioisotope or other biochemical evidence of tumour. Bronchoscopy showed apparent complete regression of the primary. Subsequently she developed brain and later extradural spinal deposits which were treated by local radiotherapy with good response. Her CEA level during this period rose steadily, to a very high level before her death. Autopsy showed widespread metastases and regrowth of the primary.

FIG. 1.—Plasma CEA levels during the course of treatment in patient M.L. (oat-cell carcinoma). The hatched areas represent: A, radiation to the primary tumour in the chest; B, radiation to cerebral metastases; C, radiation to lumbo-sacral region for extradural metastases.

Fig. 2 shows the CEA and CT levels of a 54-year-old woman treated by radiotherapy and combination chemotherapy for an oat-cell carcinoma of the bronchus. The CEA levels in this patient were normal in the beginning and remained unchanged throughout. The CT levels are of great interest; initially the level was minimally elevated and there was then a dramatic rise following radiotherapy. During follow-up for almost a year the CT levels remained undetectable, even shortly before her death from brain metastases.

Fig. 3 shows the CEA and CT levels

FIG. 2.—Plasma CEA (○——○) and calcitonin (●——●) levels during the course of treatment in patient G.I. (oat-cell carcinoma). The hatched areas represent: A, radiation to the primary tumour in the chest; B, radiation to cerebral metastases. ↑ : combination chemotherapy (cyclophosphamide, methotrexate, CCNU).

FIG. 3.—Plasma CEA (○——○) and calcitonin (●——●) levels during the course of treatment in patient W.H. (oat-cell carcinoma). The hatched areas represent: A, radiation to the primary tumour in the chest; B, radiation to hepatic metastases. ↑ : combination chemotherapy (see text).

in a 57-year-old woman with an oat-cell carcinoma of the bronchus involving local nodes and probably peribronchial tissues. Radiotherapy was started for the primary lesion (3000 rad in 10 fractions) and 5 days later the patient was complaining of nausea, vomiting and pain in the right shoulder, and on examination jaundice and tender hepatomegaly were noted. A liver scan was made and intensive chemotherapy was begun, using a combination of prednisone, 5-fluorouracil, cyclophosphamide and adriamycin and followed up a week later with bleomycin and vincristine. Symptomatic improvement ensued and the liver was noted to be smaller. Radiotherapy was given to the liver (200 rad, followed by 300 rad one month later) and chemotherapy was continued. A repeat liver scan confirmed the diminution in the size of the whole organ and of the cold areas noted on the initial scan. Similarly the chest X-ray showed almost complete resolution of the opacity in the left lung. Both CEA and CT levels were raised prior to treatment, and showed a further increase during her initial radiotherapy. Subsequently there was a marked fall in the levels of both markers (Fig. 3) as a result of chemotherapy. The patient died unexpectedly 2 months after commencing treatment. Post mortem examination revealed that viable tumour tissue was only present in the liver, where none of the nodules was greater than 2·0 cm in diameter.

### DISCUSSION

The practical importance of any circulating tumour marker depends on 3 main factors. Firstly, the frequency with which the marker is found in any population of tumour patients; secondly, a good correlation between the marker level and the mass of tumour; and thirdly, the availability of an effective treatment for the malignancy in question.

The importance of marker frequency depends on the way in which it is intended to use the information obtained. If it is intended to use the marker as a screening or diagnostic test, then it must be present in virtually 100% of cases. In this context, Concannon *et al.* (1974) concluded that CEA is of no value in bronchial carcinoma. Similarly, if the marker is to be applied as a test for disseminated disease, and thus exclude patients who are unsuitable for surgery, it must correlate very closely with the presence of metastases. Although CEA does not meet these requirements, Vincent *et al.* (1975) have presented good evidence to show that the prognosis is very poor in patients with significantly raised CEA levels who undergo surgery. In this series, 5/7 patients with initial CEA levels above 15 $\mu$g/l (Hoffmann La Roche) were dead in 5 months, and all were dead within one year.

The correlation between the level of tumour marker and the mass of tumour depends on a number of factors. As indicated in the results section, the CEA levels tended to rise as the disease advanced. However, the plasma level at any one time reflects a balance between production and degradation and it is impossible to say whether there is a precise relationship between the CEA (or CT) level and the tumour mass. The fact that the CEA levels rose in an exponential fashion in some patients (Fig. 1) would suggest that some correlation exists.

In several tumours it has been shown that a suitable marker will detect the presence of disseminated disease which it is impossible to diagnose by other methods. The most notable examples are choriocarcinoma (Bagshawe, 1967; Crawford, 1972) and testicular teratomas in which the measurement of HCG$\beta$ subunit (Cochran *et al.*, 1974; Keogh *et al.*, 1975) has been applied successfully to treatment. Undoubtedly, CEA can detect recurrence of colo-rectal cancer before it becomes clinically apparent, but this association has not had the same impact because chemotherapy is not as effective when these tumours disseminate.

It is possible to ascertain the value of a tumour marker, only when there is a sufficient variety of chemotherapeutic agents and schedules available to permit variations in treatment according to changes in levels of circulating marker substance. Previously, chemotherapy for bronchial carcinoma has not been particularly effective but, more recently, several published series have shown that the prognosis for oat-cell carcinomas may be improved by the use of more radical combinations of radiotherapy and chemotherapy (Eagan *et al.*, 1974; Johnson *et al.*, 1976; Choi and Carey, 1976; Hornback *et al.*, 1976). In the past this type of carcinoma has carried a very poor prognosis because the disease is almost always disseminated at the time of diagnosis. Nevertheless, oat-cell carcinomas can be considered to have some advantages, in that they are usually extremely responsive to irradiation and also more responsive to chemotherapy than other histological types of bronchial carcinoma. In addition, they produce a variety of tumour markers.

The data presented here indicate that knowledge of changes in tumour-marker levels could influence the choice and schedule of therapy and might effect a more favourable outcome. Thus, the case of oat-cell carcinoma of the right upper-lobe bronchus illustrated in Fig. 1 showed a marked and rapid decrease in the level of CEA with local radiotherapy. For some considerable time after completion of treatment, there was no evidence of disease other than the slightly raised CEA level. The chest X-ray, bronchoscopy and radioisotope bone scan were all normal throughout the course of the disease, until a few weeks before the patient's death. The clinical evidence of such an excellent local response would suggest that the tumour was highly radiosensitive. Given the known natural history of oat-cell tumours, chemotherapy would seem to offer the only hope of a prolonged remission. In this case, if the CEA level had been used as a guide to treatment, chemotherapy would have been instituted during the period of apparent remission. Similarly, a rising CEA (or other marker) level during one chemotherapeutic regimen should prompt a change to alternative drug combinations.

The cases in which both CEA and CT were measured revealed some interesting differences. The case of oat-cell carcinoma illustrated in Fig. 2 had normal CEA levels throughout, whereas the CT level was abnormal at the onset and showed a dramatic rise with radiotherapy, possibly due to sudden release of stored hormone from regressing tumour tissue. In this patient, chemotherapy was instituted and the CT became undetectable and remained so during follow-up for almost one year. Thus, it would seem that neither CEA nor CT had any potential as markers in this patient, despite the fact that the tumour appeared to be producing and storing CT. However, it must be remembered that the CT radioimmunoassay used in this study does not detect levels of the hormone circulating in normal individuals. The use of a more sensitive assay (Hillyard *et al.*, 1977) in this patient might have shown that the CT level was increasing, yet remaining within the normal range, as the disease progressed. In other cases, as shown in Fig. 3, both CEA and CT were elevated. In this patient with disseminated oat-cell carcinoma, a treatment programme was planned along the lines suggested by Johnson *et al.* (1976). An excellent clinical response was obtained, and this was accompanied by a well marked drop in the levels of CEA and CT.

Clearly CEA and CT are not ideal tumour markers for bronchial carcinoma, but in an individual case where the level of one or other marker is elevated initially, or subsequently rises to abnormal levels, its measurement may prove a valuable aid to therapy. Although the proportion of cases in which this occurs is low, the disease is common, and therefore CEA and CT measurements are of potential use in a large number of patients.

This would be of particular importance in oat-cell tumours, in which CEA levels are frequently elevated and CT levels are abnormal in 75% of cases. Further longitudinal studies are required in order to define more precisely the role of these tumour markers in the overall management of bronchial carcinoma.

This work was supported in part by the Cancer Research Campaign, the Medical Research Council and the Arthritis and Rheumatism Council. We wish to thank Dr J. S. Woodhead for the gift of human parathyroid hormone, Ciba-Geigy Ltd for synthetic human calcitonin and Professor A. M. Neville for the cooperation extended by his unit. MRC preparations 70/50, 71/324, 76/517, 76/507 and 75/549 were used in this study.

### REFERENCES

BAGSHAWE, K. D. (1967) Gonadotrophin Excretion, Pelvic Arteriography and Treatment in Postmolar Trophoblastic Disease. *Proc. R. Soc. Med.*, **60**, 240.

CHOI, C. H. & CAREY, R. W. (1976) Small Cell Anaplastic Carcinoma of Lung Reapraisal of Current Management. *Cancer, N.Y.*, **37**, 2651.

COCHRAN, J. S., WALSH, P. C., PORTER, J. C., NICHOLSON, T. P. & PETERS, P. C. (1974) Clinical Evaluation of Human Chorionic Gonadotrophin Levels in Men with Testicular Tumours. *Surg. Forum.*, **25**, 542.

CONCANNON, J. P., DALBOW, M. J., LIEBLER, G. A., BLAKE, K. E., WELL, C. S. & COOPER, J. W. (1974) The Carcinoembryonic Antigen Assay in Bronchogenic Carcinoma. *Cancer, N.Y.*, **34**, 184.

COOMBES, R. C., HILLYARD, C. J., GREENBERG, P. B. & MACINTYRE, I. (1974) Plasma Immunoreactive Calcitonin in Patients with Non-thyroid Tumours. *Lancet*, i, 1080.

CRAWFORD, J. W. (1972) Follow-up of Hydatidiform Mole by Radioimmunoassay of Human Chorionic Gonadotrophin. *Br. med. J.*, iv, 715.

EAGAN, R. T., MAURER, J. H., FORCIER, R. J. & TULLOH, M. (1974) Small Cell Carcinoma of the Lung: Staging, Paraneoplastic Syndromes, Treatment and Survival. *Cancer, N.Y.*, **33**, 527.

GREENBERG, P. B., DOYLE, F. H., FISHER, M. T., HILLYARD, C. J., JOPLIN, G. F., PENNOCK, J. & MACINTYRE, I. (1974) Treatment of Paget's Disease of Bone with Synthetic Human Calcitonin. Biochemical and Roentgenologic Changes. *Am. J. Med.*, **56**, 867.

HANSEN, H. J., SNYDER, J. J., MILLER, E., VANDEVOORDE, J. P., MILLER, O. N., HINES, L. R. & BURNS, J. J. (1974) Carcinoembryonic Antigen (CEA) Assay, a Laboratory Adjunct in the Diagnosis and Management of Cancer. *Hum. Path.*, **5**, 139.

HILLYARD, C. J., COOKE, T. J. C., COOMBES, R. C., EVANS, I. M. A. & MACINTYRE, I. (1977) Normal Plasma Calcitonin: Circadian Variation and Response to Stimuli. *Clin. Endocr.*, **6**, 291.

HORNBACK, N. B., EINHORN, L., SHIDNIA, H., JOE, B. T., KRAUSE, M. & FURNAS, B. (1976) Oat-cell Carcinoma of the Lung. Early Treatment Results of Combination Radiation Therapy and Chemotherapy. *Cancer, N.Y.*, **37**, 2658.

HORWITZ, H., WRIGHT, T. L., PERRY, H. & BARRETT, C. M. (1965) "Suppressive" Chemotherapy in

Bronchogenic Carcinoma. A Randomized Prospective Clinical Trial. *Am. J. Roentgenol.*, **93**, 615.

JOHNSON, R. E., BRERETON, H. D. & KENT, C. H. (1976) Small-cell Carcinoma of the Lung; Attempt to Remedy Causes of Past Therapeutic Failure. *Lancet*, ii, 289.

KEOGH, B., HRESHCHYSHYN, M. M., MOORE, R. H., MERRIN, C. E. & MURPHY, G. P. (1975) Urinary Gonadotropins in Management and Prognosis of Testicular Tumour. *Urology*, **5**, 496.

LAURENCE, D. J. R., STEVENS, U., BETTELHEIM, R., D'ARCY, D., LEESE, C., TURBERVILLE, C., ALEXANDER, P., JOHNS, E. W. & NEVILLE, A. M. (1972) Role of Plasma Carcinoembryonic Antigen in Diagnosis of Gastrointestinal, Mammary and Bronchial Carcinoma. *Br. med. J.*, iii, 605.

MACKAY, A. M., PATEL, S., CARTER, S., STEVENS, U., LAURENCE, D. J. R., COOPER, E. H. & NEVILLE, A. M. (1974) Role of Serial Plasma CEA Assays in Detection of Recurrent and Metastatic Colorectal Carcinomas. *Br. med. J.*, iv, 382.

VINCENT, R. G. & CHU, T. M. (1973) Carcinoembryonic Antigen in Patients with Carcinoma of the Lung. *J. thorac. cardiovasc. Surg.*, **66**, 320.

VINCENT, R. G., CHU, T. M., FERGEN, T. B. & OSTRANDER, M. (1975) Carcinoembryonic Antigen in 228 Patients with Carcinoma of the Lung. *Cancer, N.Y.*, **36**, 2069.